# How robust is the evidence of an emerging or increasing female excess in physical morbidity between childhood and adolescence? Results of a systematic literature review and meta-analyses

**DOI:** 10.1016/j.socscimed.2012.11.039

**Published:** 2013-02

**Authors:** Alice MacLean, Helen Sweeting, Matt Egan, Geoff Der, Joy Adamson, Kate Hunt

**Affiliations:** a MRC/CSO Social and Public Health Sciences Unit, Glasgow, UK; b University of York, UK

**Keywords:** Physical morbidity, Child, Adolescent, Gender, Review, Meta-analyses, Headache, Diabetes

## Abstract

For asthma and psychological morbidity, it is well established that higher prevalence among *males* in childhood is replaced by higher prevalence among *females* by adolescence. This review investigates whether there is evidence for a similar emerging female ‘excess’ in relation to a broad range of physical morbidity measures. Establishing whether this pattern is generalised or health outcome-specific will further understandings of the aetiology of gender differences in health. Databases (Medline; Embase; CINAHL; PsycINFO; ERIC) were searched for English language studies (published 1992–2010) presenting physical morbidity prevalence data for males and females, for at least two age-bands within the age-range 4–17 years. A three-stage screening process (initial sifting; detailed inspection; extraction of full papers), was followed by study quality appraisals. Of 11 245 identified studies, 41 met the inclusion criteria. Most (*n* = 31) presented self-report survey data (five longitudinal, 26 cross-sectional); 10 presented routinely collected data (GP/hospital statistics). Extracted data, supplemented by additional data obtained from authors of the included studies, were used to calculate odds ratios of a female excess, or female:male incident rate ratios as appropriate. To test whether these changed with age, the values were logged and regressed on age in random effects meta-regressions. These showed strongest evidence of an emerging/increasing female excess for self-reported measures of headache, abdominal pain, tiredness, migraine and self-assessed health. Type 1 diabetes and epilepsy, based on routinely collected data, did not show a significant emerging/increasing female excess. For most physical morbidity measures reviewed, the evidence broadly points towards an emerging/increasing female excess during the transition to adolescence, although results varied by morbidity measure and study design, and suggest that this may occur at a younger age than previously thought.

## Highlights

► Review of international evidence of gender-by-age differences in child and adolescent physical morbidity. ► Meta-regressions show an emerging/increasing female excess for several measures. ► An emerging/increasing male excess was seen for type 1 diabetes only. ► Findings suggest female excess physical morbidity emerges in mid-late childhood. ► Longitudinal studies commencing in childhood are needed to understand gender patterning of health.

## Introduction

It is well established that higher rates of psychological morbidity among males in childhood are replaced by higher female rates in adolescence and adulthood (Bennett, Ambrosini, Kudes, Metz, & Rabinovich, 2005; Cohen et al., 1993; Cyranowski, Frank, Young, & Shear, 2000; Ge, Conger, & Elder, 2001; Hankin & Abramson, 1999; Marcotte, Fortin, Potvin, & Papillon, 2002; Nolen-Hoeksema & Girgus, 1994; Petersen, Sarigiani, & Kennedy, 1991; Schraedley, Gotlib, & Hayward, 1999; Shibley Hyde, Mezulis, & Abramson, 2008). This pattern has also been reported for asthma prevalence (Almqvist, Worm, & Leynaert, 2008; Nicolai, Pereszlenyiova-Bliznakova, Illi, Reinhardt, & von Mutius, 2003; Postma, 2007; Sears et al., 2003; Venn, Lewis, Cooper, Hill, & Britton, 1998; Zannolli & Morgese, 1997). This paper examines whether there is evidence of an emerging or increasing female ‘excess’ in relation to other forms of physical morbidity. Identifying and appraising evidence for this pattern, particularly assessing whether it is general or specific to certain symptoms or conditions, is important, since it may enhance understanding of the apparent deterioration in some aspects of female health which begins in adolescence and continues into adulthood, and hence indicate potential ways to ameliorate gender differentials.

In the early 1990s, Sweeting (1995) conducted a narrative review of research on physical health (longstanding illness and the specific conditions asthma, diabetes mellitus, migraine and other headaches), psychological well-being and health service utilisation of children and adolescents, highlighting a ‘gender reversal’ in the distribution of ill-health during the transition from childhood to adolescence. Since then, this pattern has been widely reported, particularly in respect of psychological morbidity, and has even been described as a central feature of adolescent health in ‘a large proportion of the world's industrialised countries’ (Torsheim et al., 2006, p. 823). Attempts to explain these patterns have concentrated on psychological morbidity (Cyranowski et al., 2000; Nolen-Hoeksema & Girgus, 1994; Rutter, Caspi, & Moffitt, 2003) and have advanced similar explanations to those put forward for gender differences in adult health, such as different roles, stresses, expectations, reporting behaviours, lifestyles, as well as genetic and biological differences.

Examples of factors which might explain the ‘gender reversal’ in health beginning in early adolescence include puberty, which is associated with hormonal changes and alterations in body shape generally regarded as more positive for males (Eme, 1979; Polce-Lynch, Myers, Kilmartin, Forsmann-Falck, & Kliewer, 1998) and also with physical symptoms or conditions such as menstrual cramps (Harlow & Park, 1996), headaches and migraine (Deubner, 1977) in females. Another possible explanation is societal gender expectations (for females to be academically successful, hard-working and attractive, for males to be strong and stoic), which become more differentiated during this life stage (MacLean, Sweeting, & Hunt, 2010; West & Sweeting, 2003), potentially leading to differences in the experience of psychosocial stressors and impacting on illness behaviours such as symptom reporting. Gender differences in lifestyle, for example higher levels of physical activity in adolescent males (Coleman & Schofield, 2001), might also contribute to gender differences in adolescent health. The relatively few studies which have specifically examined, or attempted to account for the gender reversal in relation to physical morbidity (Angold, Costello, Erkanli, & Worthman, 1999; Eminson, Benjamin, Shortall, Woods, & Faragher, 1996; Haugland, Wold, Stevenson, Aaroe, & Woynarowska, 2001; MacLean et al., 2010; Sweeting, West, & Der, 2007) have considered such explanations, but fully accounting for these patterns has proved difficult. In addition, a cross-national study of 11–15-year olds from a range of European and North American countries found that the size of gender differences in adolescent health varied between-countries and was associated with each country's male:female life expectancy ratio, suggesting that macro-social contextual factors might also impact on gender differences in adolescent health (Torsheim et al., 2006).

Contrasting with the research focus on an emerging or increasing female excess in psychological distress, no reviews have, to our knowledge, been conducted with the aim of systematically investigating the extent to which there is evidence of an emerging or increasing female excess in relation to a range of *physical* morbidity measures. There are a number of reasons for conducting such a review. Firstly, it should enable the synthesis and appraisal of evidence in relation to changing gender differences in health between childhood and adolescence in physical morbidity and, in turn, allow a judgement about the robustness of evidence for an emerging/increasing female excess. Secondly, it should shed light on whether this pattern is found for physical morbidity generally or is specific to certain symptoms or conditions. Thirdly, it could highlight new areas for investigation to further understandings of the aetiology of gender differences in health. Fourthly, it should ensure that future primary research in this area avoids replication and is informed by the ‘best available evidence’ (Bambra, 2011) of the gender-by-age patterning of physical morbidity during childhood and adolescence. Finally, it may inform strategies and interventions that aim to prevent or reduce gender inequalities in health.

**Fig. 1:**
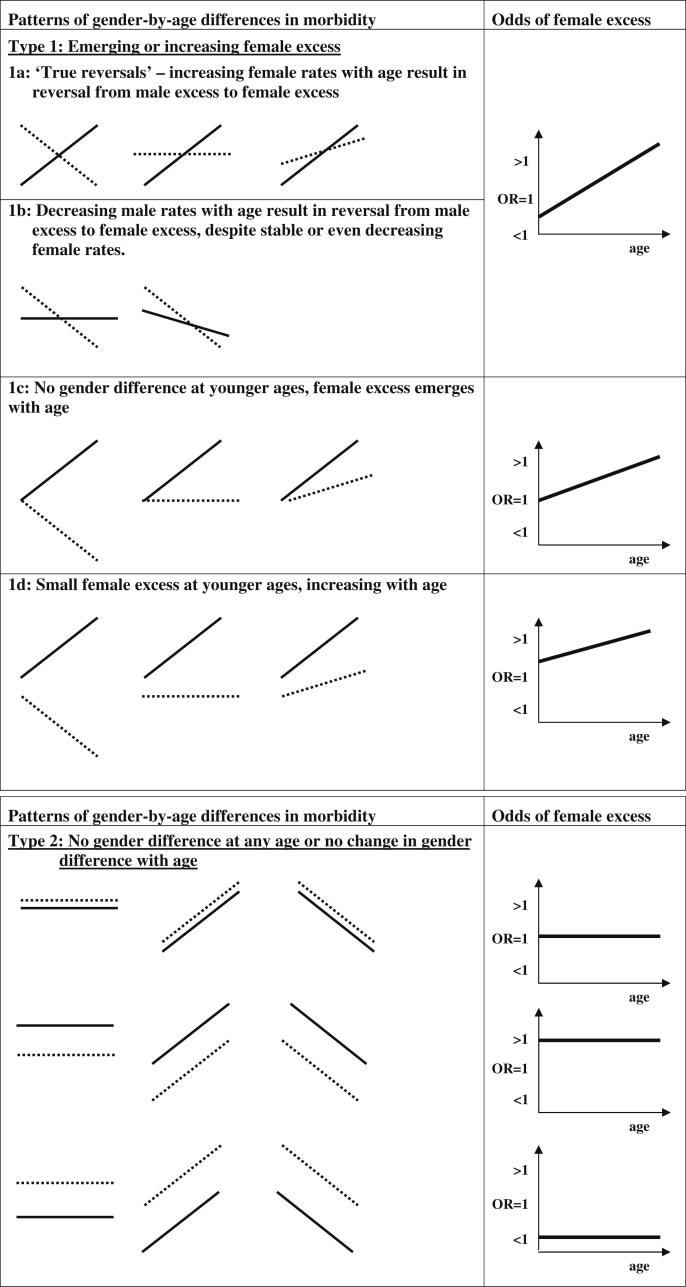

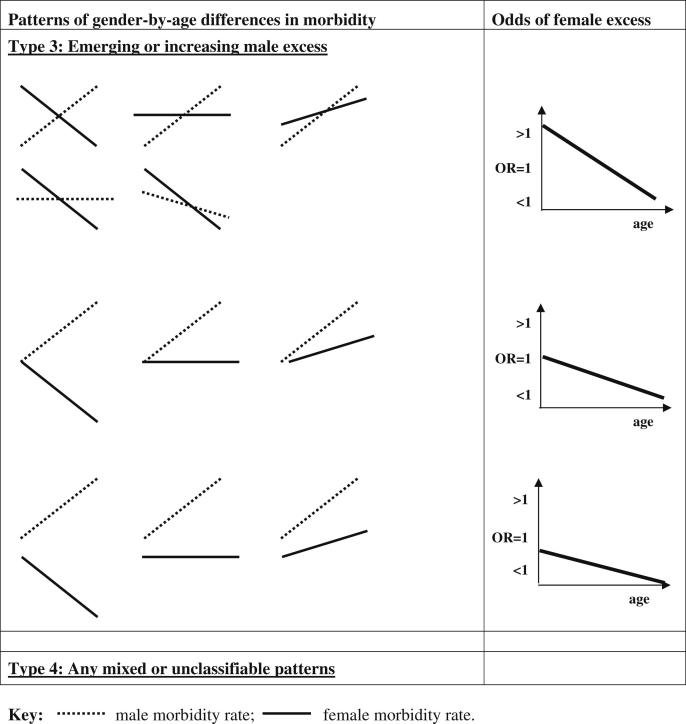
Patterns of gender-by-age differences in morbidity and associated odds of female excess morbidity.

The identification of ‘reversals of fortune’ within Sweeting's (1995) earlier review might suggest a simple switch from excess male morbidity in childhood to excess female morbidity by mid adolescence. In attempting to conceptualise patterns of gender-by-age differences in morbidity more critically and comprehensively for this review, we represented diagrammatically a series of plausible patterns. These are described below and shown in Fig. 1. Diagrams in the left-hand column schematically represent possible male and female morbidity rates from mid-late childhood to mid adolescence, whilst those in the right-hand column represent the associated odds of female excess morbidity over the same life stage.

‘Type 1 reversals’ represent variations on an emerging or increasing female excess. They can originate from: a) a male excess reversing to a female excess (via increasing female rates with age accompanied by decreasing male rates, stable male rates or male rates which increase with age, but less steeply than those of females); b) decreasing male rates with age while female rates remain stable or decrease less steeply; c) an emerging female excess from a point of no gender difference; d) an existing small female excess at younger ages which increases with age. In respect of these patterns the odds of morbidity among females compared to males would start from below (‘Types 1a’ and ‘1b’), at (‘Type 1c’) or above unity (‘Type 1d’) and increase with age. It should be noted that the specific ages examined will, at least to some extent, determine the pattern observed. For example, a male excess reversing to female excess pattern might appear as an emerging or increasing female excess if initial health measures were obtained at older ages.

‘Type 2’ patterns represent either no gender difference or no change in the gender difference with age. ‘Type 3’ represents variations on an emerging *male* excess; the patterns are the reverse of those in Type 1. Finally, ‘Type 4’ represents any mixed or unclassifiable patterns, for example an increasing then decreasing male rate combined with a stable female rate. Due to their potential complexity and reduced relevance from the point of this review, ‘Type 4’ patterns are not shown diagrammatically.

## Aims

The current review uses systematic methods to investigate the gender-by-age patterning of a range of physical morbidity measures across childhood and adolescence to identify the extent to which there is evidence of an emerging or increasing female excess in physical morbidity over this life stage. We focus on more general measures of physical health (self-assessed health) together with a number of common symptoms (abdominal pain, back pain, dizziness, sleeping problems/tiredness and headache) and conditions (migraine, diabetes mellitus and epilepsy). We are aware that distinguishing certain symptoms as ‘physical’ rather than ‘psychological’ or ‘malaise’ is necessarily somewhat arbitrary (Hunt & Annandale, 1993). All the symptoms included in this review could reflect organic (physical) disease and/or a substantial psychological component (Pennebaker, 1982).

Our objectives, in terms of the PICOS statement (Moher, Liberati, Tetzlaff, & Altman, 2009), were to identify, appraise and review studies with the following characteristics:

**Population**: males and females aged 4–17 years;
**Intervention**: none
**Comparator**: gender and age (at least two age groups);
**Outcome**: gender patterning, by age, in measures of physical morbidity;
**Study design**: longitudinal, cross-sectional and repeat cross-sectional studies (including analysis of study-specific data or routinely collected data).

## Methods

We conducted a systematic review, which we have designed and reported in line with PRISMA (Preferred Reporting Items for Systematic Reviews and Meta-Analyses) guidelines (Moher et al., 2009). The protocol for this review is available (Egan, MacLean, Sweeting, & Hunt, 2012).

### Data sources and search strategy

We searched five electronic databases (Medline, Embase, the Cumulative Index to Nursing and Allied Health Literature (CINAHL), PsycINFO, and the Education Resources Information Center (ERIC)) for studies published in English between 1992 and the date of search (April 2010). As it was our intention to update Sweeting's (1995) narrative review and to focus on contemporary evidence of gender differences in health, we aimed to identify literature published since 1992.

We searched each database using subject headings and keywords specifying both general health measures (Egan et al., 2012), as well as some of the most common childhood and adolescent self-report symptoms and chronic non-congenital/perinatal conditions (Coleman & Schofield, 2001; Starfield, 1991). Searches were tailored to specific databases but included the following terms: adolescent health; attitude to health; child health; general health questionnaire; health; health attitudes; health complaints; health status; health status indicators; health survey; morbidity; self-report; symptoms; well-being; wellness; diabetes mellitus; epilepsy; asthma; headache; headache disorders, primary; migraine; primary headache. The full search strategies are available in Appendices 1 and 2 of the research protocol. Although asthma was originally included in the specific search terms, once papers relating to asthma were extracted it became clear that several reviews of gender differences in asthma had been conducted (Almqvist et al., 2008; Postma, 2007; Zannolli & Morgese, 1997). Asthma was therefore excluded from our review to avoid repeating this effort.

### Study selection

We wanted to look at how gender differences in prevalence (generally over past month or past year) and incidence rates (for type 1 diabetes and epilepsy) may change with age. We included quantitative studies which presented prevalence and incidence data on general and specific physical morbidity measures for children under age 18, by gender for at least two age groups. Studies presenting data in age-bands wider than five years were excluded because we felt such broad age-bands would make it difficult to interpret how age was associated with the (changing) gendered patterning of health measures. Studies were excluded where we could not distinguish between longstanding and recent illness (e.g. included only lifetime prevalence data). We included studies that involved self-reported health measures and/or medical examinations by health professionals, but excluded evidence of measures that relied on proxy data, such as parents' or teachers' reports. As a result, we excluded much of the evidence relating to babies and toddlers (children considered too young to self-report). Studies of those aged 18 years and over were classed as adult studies and also excluded. Studies of young people or adults including a majority of participants within our age-range (e.g. 15–19 years) were included. Studies from current EU countries as well as the USA, Canada, Australia and New Zealand were included. Studies from all other countries were excluded on the basis that differences in wider contextual factors (e.g. levels of development, deprivation, political stability) may impact not only upon rates of experienced physical morbidity but also on the collection and reporting of morbidity data which may in turn prevent useful comparisons across countries. We also excluded studies which: presented data in relation to either males only or females only, or for both males and females for only one age group; employed only qualitative data collection and analysis methods; or presented data on health behaviours or symptoms resulting from various health behaviours (e.g. muscle pain following physical activity). Studies which presented data only on injuries, accidents, or dental health were also excluded because our primary interests were symptoms and conditions.

**Fig. 2:**
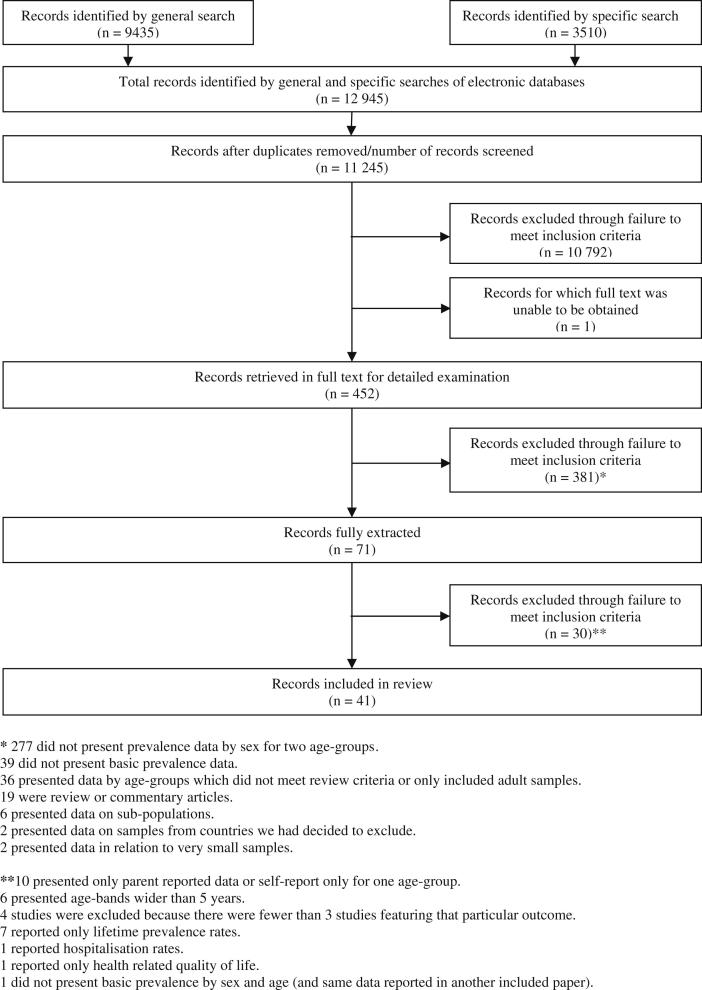
Study selection.

As illustrated in Fig. 2, the initial searches generated 11 245 titles (after removing duplicates). One reviewer (AM) screened all titles and abstracts to exclude references which were obviously irrelevant or failed to meet inclusion criteria. Where there was any uncertainty about whether studies should be excluded, two additional reviewers (KH and HS) screened the titles and abstracts and a decision was made after discussion. Full text versions of 452 studies were obtained for a second stage of screening and, after closer inspection of each study, a further 381 failed to meet our inclusion criteria. The remaining 71 were fully extracted of which 30 were subsequently excluded. Thus 41 studies were deemed relevant and of sufficient scientific quality for inclusion. It should be noted that only three studies (Santinello, Vieno, & De Vogli, 2009; Sweeting & West, 2003; Torsheim et al., 2006) explicitly stated an intention to explore the hypothesis that prevalence rates would reverse from being higher in males at younger ages to being higher in females in adolescence.

### Data extraction

After the second screening, one reviewer (AM) extracted data for the 71 remaining studies. Three reviewers (KH, HS and ME) independently extracted a random sample of 10 papers each. Although comparisons of these extraction forms generally showed a high level of agreement between reviewers, any discrepancies were discussed and minor changes made to seven of the sample of 30 extraction forms. The following data were extracted: (1) publication details: author; title; journal; date; primary focus; stated aims; (2) focus on emerging/increasing female excess, defined as: mention (or not) of sex/gender differences/similarities/‘gender reversal’ in introduction, results or discussion; explanations offered for changes in gender differences with age; (3) study details: methods; sample (source, size, age-range and age groups, representativeness, response rate/completeness); primary outcomes; questions/instruments; (4) any figures for health measures by gender and age (e.g. prevalence rate/incidence rate, both adjusted and unadjusted figures, extracted as reported in each paper (means, OR, RR etc.) with as much detail as possible (95% confidence intervals, chi-square etc.)).

### Critical appraisal

Many appraisal tools used in systematic reviews have been developed to appraise randomised control trials or other study designs used to evaluate intervention effects (although appraisal tools for other types of study, such as case control and cohort studies are also available (Wells et al., 2000)). To ensure our critical appraisal was tailored to the study designs relevant to this review (including cross-sectional studies and routine data analysis), we adapted an existing critical appraisal tool developed for use across a variety of study designs (Thomson, Thomas, Sellstrom, & Petticrew, 2009). Studies were assessed against the following potential sources of bias: sample size; multi-site design; number of age groups presented; selection bias; outcome measurement; analysis/data reporting, and (longitudinal studies only) attrition rate. These are described in detail in the review protocol (Egan et al., 2012). Studies were given an indicative score for overall quality. This was calculated by summing the grades for each appraisal criterion (highest grade = 2; middle = 1; lowest = 0). Longitudinal studies were scored out of a maximum of 14 (because they had an extra appraisal criterion: attrition); other studies were scored out of 12. All eligible studies were critically appraised by AM and checked by ME, with any differences resolved by discussion. Appraisal scores are presented in supplementary Table 1.

### Data analysis

The summary measure chosen for prevalence data was the odds ratio (OR) for a female excess and for the incidence data the incidence rate ratio (IRR), again with males as the reference group. If these measures and their confidence intervals could not be calculated from the data within any paper, authors were approached to provide additional data. Continuous data from health-related quality of life scores (Solans et al., 2008) were combined with the data on categorical self-assessed health by reversing the scores so that high scores represented poorer health and converting them to ORs using the method proposed by Chinn (2000).

For a formal test of a significant change in gender patterning with age, we performed random effects meta-regressions of the female excess on age. For these analyses the outcome was the logged OR (or IRR) of a female excess; age was taken as the mean age of the age group or the midpoint of narrow age-bands and study weights were calculated according to the method of Der Simonian and Laird (1986) based on the inverse variance of the study logged OR (IRR). For longitudinal studies, the sample sizes were divided by the number of waves in the study. The results are presented in table form as regression coefficients together with their equivalent ORs (IRRs), and as meta-regression plots. Where there is a significant effect of age, the table also reports the ‘cross-over age’, at which the female excess is estimated to begin. Where the regression coefficient is non-significant, (i.e. the pattern of female excess does not change with age) it is appropriate to report the summary ORs (IRRs); we do this in the text.

In addition to the overall summaries in respect of each morbidity measure resulting from the meta-regressions, it is possible to infer statistically significant age-related changes in gender patterning within any individual study if the 95% CIs for the ORs/IRRs for a female excess at different ages do not overlap, although this is a somewhat conservative criterion (Cumming, 2009; Wolfe & Hanley, 2002). Odds ratios/IRRs and (with the exception of two studies (Gordon, Dooley, & Wood, 2004; Karvonen, Pitkaniemi, & Tuomilehto, 1999)) associated 95% CIs were therefore also tabulated, and described in respect of which of the ‘reversal types’ they best fit (‘Type 1’ – emerging or increasing female excess; ‘Type 2’ – no gender differences or no changes in gender differences; ‘Type 3’ – emerging or increasing male excess; ‘Type 4’ mixed or unclassifiable). For the one study where ORs could not be calculated, we present descriptive results as reported by its authors (Rhee, Miles, Halpern, & Holditch-Davis, 2005).

All extracted data, critical appraisal scores and allocation of physical morbidity measures to a ‘reversal type’ have been conducted by one author and checked by at least one other author.

## Results

### Description of studies

Although the review includes 41 ‘studies’ (Fig. 2), some of these produced multiple findings by including more than one health outcome, and/or sampling more than one population and presenting separate results by gender and age for each population. In addition, five studies consisted of separate analysis of the multi-national repeat cross-sectional Health Behaviour in School-aged Children (HBSC) Survey (Cavallo et al., 2006; Haugland et al., 2001; Meland, Haugland, & Breidablik, 2007; Santinello et al., 2009; Torsheim et al., 2006). Three of these did not include common data (Haugland et al. (2001) data on symptoms from Finland, Norway, Poland and Scotland obtained in 1993–1994; Meland et al. (2007) – data on self-assessed health from Norway obtained in 1997–1998; and Torsheim et al. (2006) – data on symptoms from 29 countries obtained in 1997–1998). However two HBSC studies (Cavallo et al., 2006) (31 countries) and (Santinello et al., 2009) (Italy only), include headache data from the same country and survey wave (2001–2002). Results from the latter are therefore included in our descriptive results, but excluded from the meta-regression relating to headache.

The characteristics and main results of all 41 studies are described in supplementary Table 1. Five of these studies presented longitudinal survey data (Grimmer, Nyland, & Milanese, 2006; Laaksonen et al., 2010; Larsson & Sund, 2005; Palacio-Vieira et al., 2008; Sweeting & West, 2003), 26 (repeat) cross-sectional survey data (Bigal et al., 2007; Bisegger et al., 2005; Cavallo et al., 2006; Gadin & Hammarstrom, 2000; Gordon et al., 2004; Haugland et al., 2001; Heinrich, Morris, & Kroner-Herwig, 2009; Holmberg & Hellberg, 2007; Jorngarden, Wettergen, & von Essen, 2006; Kujala, Taimela, & Viljanen, 1999; Laurell, Larsson, & Eeg-Olofsson, 2004; Leonardsson-Hellgren, Gustavsson, & Lindblad, 2001; Lundqvist, Clench-Aas, Hofoss, & Bartonova, 2006; Mavromichalis, Anagnostopoulos, Metaxas, & Papanastassiou, 1999; Meland et al., 2007; Mortimer, Kay, & Jaron, 1992, 1993; Ostberg, Alfven, & Hjern, 2006; Petersen, Bergstrom, & Brulin, 2003; Ravens-Sieberer, Erhart, Wille, Bullinger, & Bella Study Group, 2008; Rhee et al., 2005; Santinello et al., 2009; Sleskova, Salonna, Madarasova Geckova, van Dijk, & Groothoff, 2005; Sundblad, Saartok, & Engstrom, 2007; Torsheim et al., 2006; Wedderkopp, Leboeuf-Yde, Andersen, Froberg, & Hansen, 2001), and 10 routinely collected data (Beilmann et al., 1999; Carle et al., 2004; Casu, Pascutto, Bernardinelli, Sardinian IDDM Epidemiology Study Group, & Songini, 2004; Christensen et al., 2007; Cinek et al., 2000; Cotellessa et al., 2003; Freitag, May, Pfafflin, Konig, & Rating, 2001; Karvonen et al., 1999; Michalkova, Cernay, Dankova, Rusnak, & Fandakova, 1995; Skordis et al., 2002), such as clinic/hospital statistics.

The median critical appraisal score was 10 out of a possible 14 for longitudinal studies (range 6–12); 8 (out of a possible 12) for (repeat) cross-sectional surveys (range 4–11); and 11 (out of 12) for studies presenting analyses of routinely collected data (range 9–12). These three median scores are used as benchmarks to compare the relative quality of included studies within each type. Thus we describe longitudinal studies, cross-sectional surveys and routine data analysis studies scoring >10, >8 and >11 respectively as ‘higher-scoring’. Twelve (29%) of the included studies were consequently described as ‘higher-scoring’ (Beilmann et al., 1999; Carle et al., 2004; Cavallo et al., 2006; Haugland et al., 2001; Heinrich et al., 2009; Mavromichalis et al., 1999; Ostberg et al., 2006; Petersen et al., 2003; Santinello et al., 2009; Sundblad et al., 2007; Sweeting & West, 2003; Torsheim et al., 2006).

The results are grouped as follows: self-assessed health, symptoms (abdominal pain, back pain, dizziness, sleeping problems/tiredness, headache) and conditions (migraine, type 1 diabetes, epilepsy). For each morbidity measure we present the results of the meta-regressions followed by more details of individual studies. Results in respect of the first measure, self-assessed health, are described in detail to facilitate comprehension of the remaining health measures considered. These latter results are briefer and generally restricted in the text to ‘higher-scoring’ studies (results relating to all studies, ‘higher’ and ‘lower-scoring’, are included within the tables). The text states when a study is ‘higher-scoring’; any studies not identified as such are ‘lower-scoring’.

### Self-assessed health

**Fig. 3:**
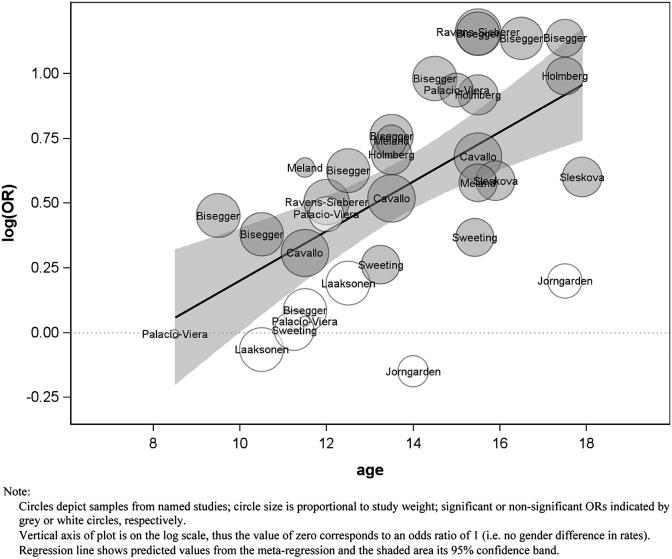
Plot of meta-regression testing the hypothesis of a changing female excess in self-assessed health with age.

We identified three longitudinal and seven cross-sectional studies measuring self-assessed health among males and females across the transition from childhood to adolescence. The result of the meta-regression testing the hypothesis of a changing female excess in self-assessed health with age is shown in the first row of Table 1. The regression coefficient (*B*) is the change in the log OR of a female excess for one year of age. This is statistically significant and positive, indicating an increasing probability of poor self-assessed health among females, relative to males, with age. The exponentiated (anti-logged) value of the coefficient is the OR, again per year of age and this (OR = 1.10) shows that the relative odds of poor self-assessed health increase at a rate of 10% per year within the ages included in the studies reviewed (clearly it would be unwise to extrapolate these trends beyond the data available). Where there is a significant effect of age, Table 1 also shows the estimated ‘cross-over age’, that is, the age at which the female excess is estimated to begin. This was 7.9 years for self-assessed health, although some caution should be exercised here as this value lies slightly outside the range of the data (see Fig. 3).

The meta-regression plot for self-assessed health depicts samples from named studies (Fig. 3). The size of the circles is proportional to the study weight. As the vertical axis of the plot is on the log scale, the value of zero corresponds to an OR of 1 (i.e. no gender difference in rates), and a reference line is drawn at this point. Significant or non-significant ORs are indicated by grey or white circles, respectively. Grey circles above this reference line indicate a significant female excess, those below the line a significant male excess. The regression line shows the predicted values from the meta-regression and the shaded area its 95% confidence band.

Further details of the 10 self-assessed health studies included in the meta-regression are shown in Table 2. Five presented ORs (or data allowing their calculation) of poor categorical self-assessed health among females compared with males, two of which (both ‘higher-scoring’) show evidence of statistically significant gender-by-age interactions. These were a cross-sectional study (Cavallo et al., 2006) reporting an increasing female excess of fair/poor (vs. good/excellent) health between ages 11 (OR 1.36) and 15 (OR 1.97), and a longitudinal study (Sweeting & West, 2003) in which the female excess of fairly/not good (vs. good) health increased from OR of 1.01 at 11 to 1.44 at age 15. The other three cross-sectional studies of poor categorical self-assessed health shown on Table 2 (Holmberg & Hellberg, 2007; Meland et al., 2007; Sleskova et al., 2005) did not find statistically significant gender-by-age interactions, although the data presented by Holmberg and Hellberg (2007) suggest an increasing female excess.

The other five self-assessed health studies reported on physical subscale or summary scores from health-related quality of life (HRQoL) measures. As noted above, these have been converted into ORs to aid comparability with the categorical measures. Two of these were longitudinal studies (Laaksonen et al., 2010; Palacio-Vieira et al., 2008) both of which, together with one cross-sectional study (Jorngarden et al., 2006) showed trends towards an increasing female excess, but with overlapping CIs. The other two cross-sectional studies reporting ‘physical’ HRQoL results report findings that fit the pattern of an emerging female excess with non-overlapping CIs (Bisegger et al., 2005; Ravens-Sieberer et al., 2008).

### Symptoms

#### Abdominal pain

Twelve (one longitudinal, 11 cross-sectional) studies reported on prevalence of abdominal pain in males and females across the transition from childhood to adolescence. One cross-sectional study (Haugland et al., 2001) presented separate analyses for surveys carried out in four countries, thus 15 samples relating to abdominal pain were available, 14 of which could be included in the meta-regression (Table 3).

**Fig. 4:**
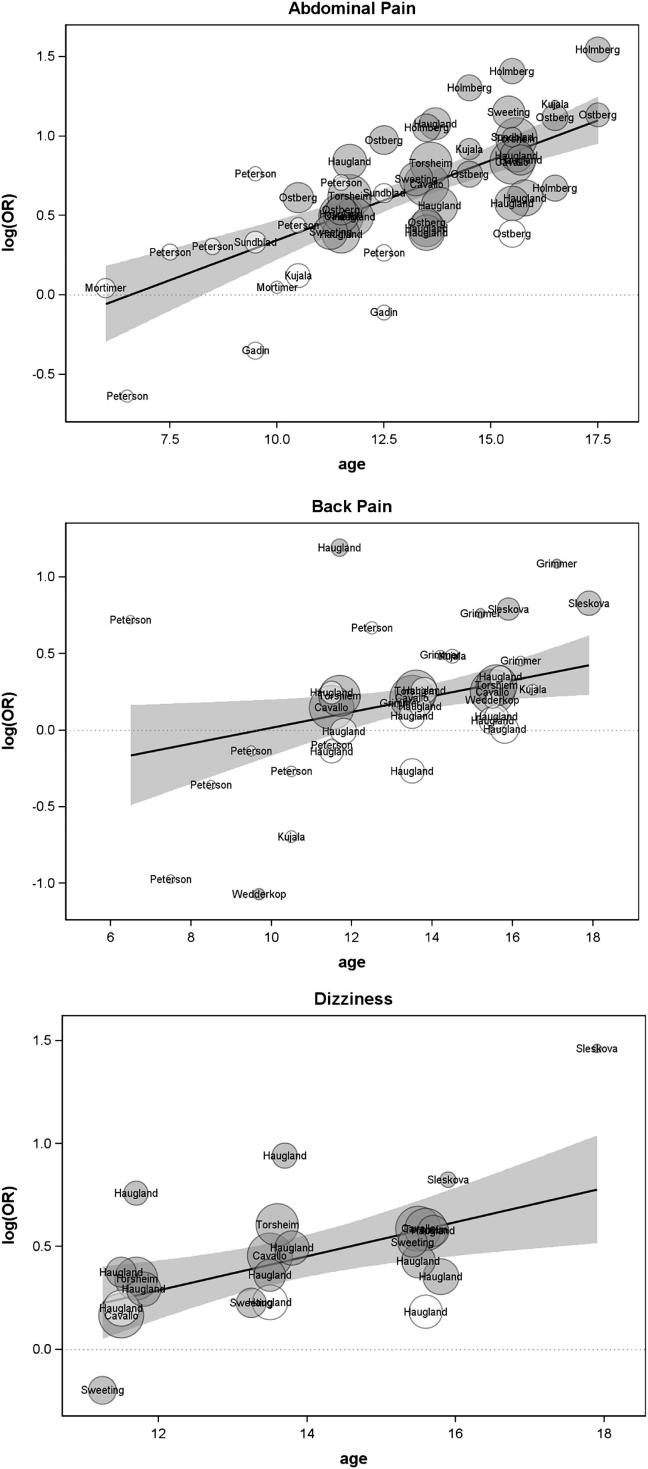

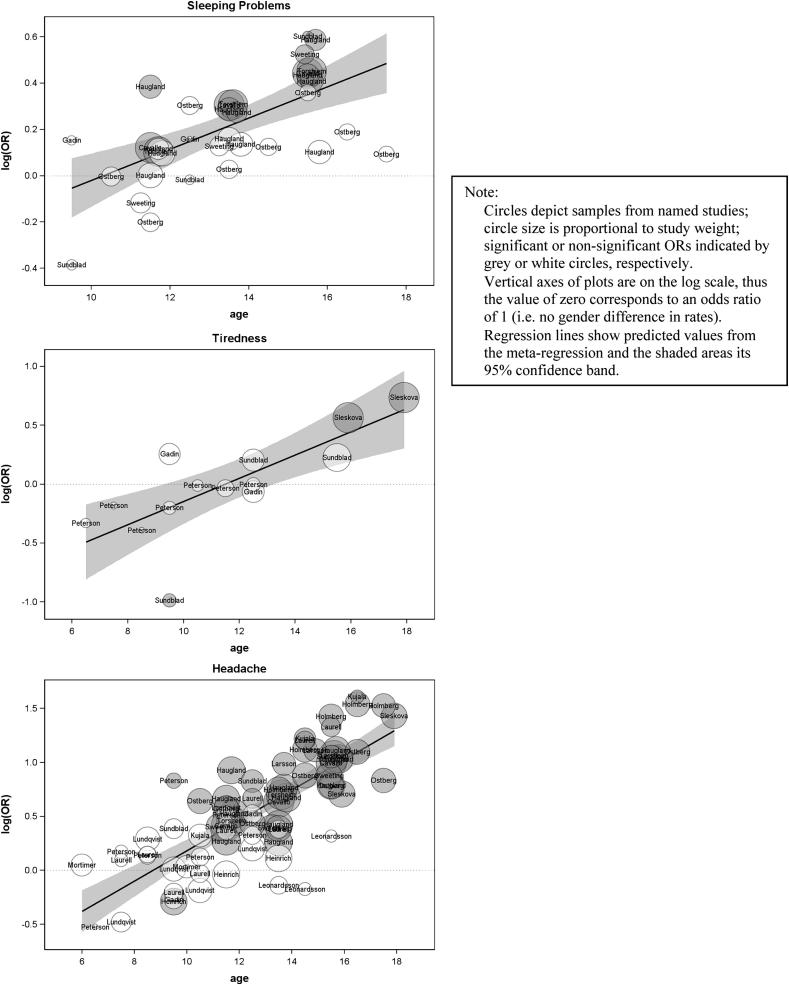
Plot of meta-regression testing the hypothesis of a changing female excess in symptoms (abdominal pain, back pain, dizziness, sleeping problems, tiredness, headache) with age.

The result of the meta-regression in respect of abdominal pain is shown in Table 1 (second row) and the associated meta-regression plot in Fig. 4. The significant, positive regression coefficient and associated OR = 1.11 indicate that the probability of abdominal pain for females, relative to males increases significantly, and at a rate of 11% per year of age within the ages included in the reviewed studies. The age at which the female excess in abdominal pain is estimated to begin was 6.6 years.

Table 3, which details the individual studies, shows analyses from three (all ‘higher-scoring’) abdominal pain studies provided evidence of significant ‘Type 1’ gender-by-age interactions. These were two different HBSC surveys, conducted in around 30 European and North American countries (Cavallo et al., 2006; Torsheim et al., 2006) and a longitudinal study Sweeting and West (2003); each of these studies included data from 11, 13 and 15-year olds. Findings from the other ‘higher-scoring’ abdominal pain studies were either suggestive of a ‘Type 1’ pattern but with overlapping 95% CIs for the oldest and youngest age groups (Haugland et al., 2001 (Finland and Scotland); Ostberg et al., 2006; Sundblad et al., 2007) or showed a mixed (‘Type 4’) pattern (Haugland et al., 2001 (Poland and Norway); Petersen et al., 2003).

#### Back pain

We identified one longitudinal and seven cross-sectional studies focussing on back pain, including one with four separate country analyses (Table 3). While five of these studies measured ‘back pain’ generally (Cavallo et al., 2006; Haugland et al., 2001; Petersen et al., 2003; Sleskova et al., 2005; Torsheim et al., 2006), three measured ‘low back pain’ (Grimmer et al., 2006; Kujala et al., 1999; Wedderkopp et al., 2001).

The result of the meta-regression (Table 1, third row) and associated meta-regression plot (Fig. 4) indicates that the probability of back pain for females, relative to males increases significantly, at a rate of 5% per year of age within the ages included in the reviewed studies. The age at which the female excess in back pain is estimated to begin was 9.7 years.

Seven back pain analyses were from ‘higher-scoring’ studies (Table 3). One of these (Cavallo et al., 2006) provided evidence of statistically significant ‘Type 1’ gender-by-age interactions, another (Torsheim et al., 2006) was suggestive of the ‘Type 1’ pattern and three showed a ‘Type 4’ (mixed) pattern (Haugland et al., 2001 (results for Finland and Scotland); Petersen et al., 2003). Finally, two analyses reported by Haugland et al. (2001), suggested ‘Type 3’ (emerging male excess) patterns, with decreasing odds of a female excess in back pain between ages 11 and 15 in Norway and Poland, but both with overlapping CIs.

#### Dizziness

The meta-regression results from eight samples with data relating to dizziness (Table 1) and associated meta-regression plot (Fig. 4) indicates that the probability of a female excess in dizziness increases significantly and at a rate of 9% per year of age within the ages included in the reviewed studies. The female excess in dizziness is estimated to begin at 8.5 years.

Table 3 summarises results from nine analyses of dizziness (from six studies). Four of these studies were ‘higher-scoring’. Of these, three (Cavallo et al., 2006; Sweeting & West, 2003; Torsheim et al., 2006) showed significantly increasing ORs of self-reported dizziness amongst females compared with males between ages 11 and 15. However, the fourth (Haugland et al., 2001) provided no evidence of significant gender-by-age interactions in any of its four country-specific analyses.

#### Sleeping problems/tiredness

Table 3 reports ten analyses relating to sleeping problems and five relating to tiredness.

The meta-regression results (Table 1) and associated meta-regression plots (Fig. 4) indicate that the probabilities for females, relative to males increase significantly, at a rate of 7% (sleeping problems) and 10% (tiredness) per year of age, within the ages included in the reviewed studies. The female excess in these symptoms is estimated to begin at 10.3 years for sleeping problems and 11.5 years for tiredness.

Of the 15 separate analyses, 11 were from ‘higher-scoring’ studies. Six of these provided statistically significant evidence of a ‘Type 1’ pattern, five in respect of sleeping problems (Cavallo et al., 2006; Haugland et al., 2001 (Scotland results); Sundblad et al., 2007; Sweeting & West, 2003; Torsheim et al., 2006) and one in respect of tiredness (Sundblad et al., 2007). None of the other ‘higher-scoring’ sleeping problems/tiredness analyses showed significant changes in gender differences with age.

#### Headache

We identified two longitudinal and 17 cross-sectional studies measuring prevalence of headache (22 sets of analyses in total - Table 3). The meta-regression results (Table 1) and associated meta-regression plot (Fig. 4) indicate that out of all the included physical morbidity measures, the probability of a female excess in headache shows the steepest increase with age (15% per year of age within the ages included in the reviewed studies). The female excess in headache is estimated to begin at 8.7 years.

Twelve of the individual headache analyses came from ‘higher-scoring’ studies (Table 3), of which six provided statistically significant evidence of a ‘Type 1’ pattern (Cavallo et al., 2006; Haugland et al., 2001 (Finland and Scotland results respectively); Santinello et al., 2009; Sweeting & West, 2003; Torsheim et al., 2006). Of the remaining analyses from ‘higher-scoring’ studies, two showed ‘Type 1’ trends (Heinrich et al., 2009; Sundblad et al., 2007) and four a mixed (‘Type 4’) pattern (Haugland et al., 2001 (Norway and Poland); Ostberg et al., 2006; Petersen et al., 2003).

### Conditions

#### Migraine

**Fig. 5:**
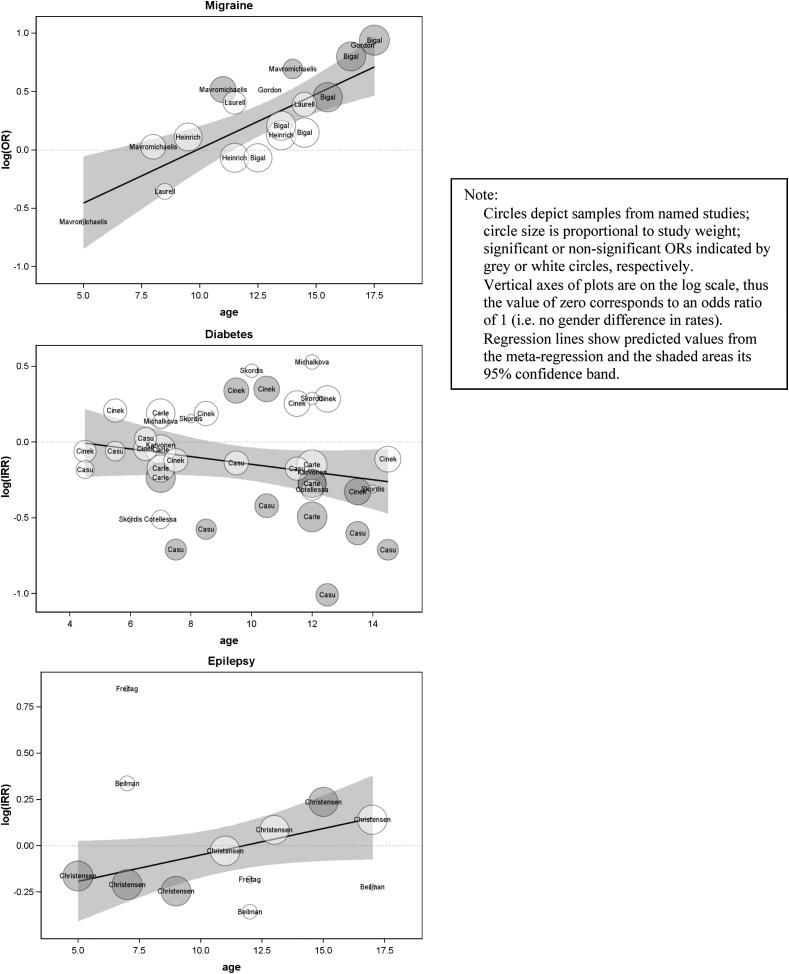
Plot of meta-regression testing the hypothesis of a changing female excess in conditions (migraine, type 1 diabetes, epilepsy) with age.

We identified five studies measuring prevalence of migraine (self-reported according to internationally recognised criteria). The meta-regression (Table 1, Fig. 5) indicated that the probability of migraine among females, relative to males, increases significantly, and at a rate of 10% per year of age within the ages included in the reviewed studies, and that the female excess in migraine is estimated to begin at 9.9 years.

Table 4 details the individual migraine studies, two of which were ‘higher-scoring’. One of these showed trends towards a ‘Type 1’ pattern in past year migraine to 1988 IHS criteria (Mavromichalis et al., 1999, higher scoring). In the other, there was little evidence of gender differences in past six months migraine (2004 IHS criteria) at any age (Heinrich et al., 2009).

#### Type 1 diabetes

Seven studies reporting incidence of Type 1 diabetes were identified, all based on routine data. Since one (Carle et al., 2004) presented findings for data from four separate Italian regions, the diabetes studies comprise ten separate sets of findings. The meta-regression (Table 1, Fig. 5) indicated no increase with age in the probability of diabetes incidence among females, relative to males. Indeed, there was a non-significant trend in the opposite direction (i.e. an increasing probability of diabetes incidence among males, relative to females, with age – a ‘Type 3’ pattern) within the ages included in the reviewed studies. Since the non-significant regression coefficient indicates no change with age in the pattern of female excess, it is appropriate to report the summary IRR (95% CI). This was 0.87 (0.79–0.97), indicating excess incidence of diabetes among males within the age groups included in the studies reviewed here.

Table 5, which details the individual diabetes studies, shows that in no sample did the female excess in incidence differ significantly between age groups. However, six of the ten analyses showed trends towards an increasing or emerging male excess of diabetes incidence with age, including all four samples from the only ‘higher-scoring’ diabetes study (Carle et al., 2004; Casu et al., 2004; Karvonen et al., 1999).

#### Epilepsy

In relation to epilepsy incidence, we identified three studies, all based on routine data (Table 5). The meta-regression (Table 1, Fig. 5) indicated a non-significant increase with age in the probability of epilepsy incidence among females, relative to males. The summary IRR (95% CI), which it is therefore appropriate to report, was 0.98 (0.86–1.11), indicating no clear gender difference in epilepsy incidence within the age groups included in the studies reviewed here.

This result is based on a small number of studies, only one of which was ‘higher-scoring’. Table 5 which details the epilepsy studies, shows that the female:male IRR in the higher scoring study (Beilmann et al., 1999) showed trends towards an emerging male excess. However, analysis of a much larger sample (Christensen et al., 2007) showed a trend towards an emerging female excess.

## Sensitivity analysis

A sensitivity analysis was conducted to assess the impact of including only one wave from each of the longitudinal studies. The meta-regressions were repeated selecting each wave from the longitudinal studies in turn. Headache and self-assessed health had data from more than one longitudinal study and in those cases all possible combinations of waves were generated (6 for headache and 24 for self-assessed health). The coefficients from these repeated analyses were then averaged. The resulting values were little changed – the largest differences being for dizziness (*B* = 0.074); self-assessed health (*B* = 0.086) and sleep problems (*B* = 0.063).

## Discussion

Past research and systematic reviews of psychological morbidity and asthma have reported robust evidence that higher male prevalence rates in childhood are replaced by an emergence of higher prevalence among adolescent females (Almqvist et al., 2008; Bennett et al., 2005; Cohen et al., 1993; Cyranowski et al., 2000; Ge et al., 2001; Hankin & Abramson, 1999; Marcotte et al., 2002; Nicolai et al., 2003; Nolen-Hoeksema & Girgus, 1994; Petersen et al., 1991; Postma, 2007; Schraedley et al., 1999; Sears et al., 2003; Shibley Hyde et al., 2008; Venn et al., 1998; Zannolli & Morgese, 1997). For example, two reviews of gender and age differences in asthma prevalence and incidence during childhood and adolescence reported that asthma is more common in males before age ten and in females by the mid-teen years (Almqvist et al., 2008; Zannolli & Morgese, 1997). Studies have also reported this pattern for other forms of physical morbidity (Eiser, Havermans, & Eiser, 1995; Eminson et al., 1996; Haugland et al., 2001; Hetland, Torsheim, & Aaro, 2002; Klepp, Aas, Maeland, & Alasker, 1996; Sweeting & West, 2003; Torsheim et al., 2006). However, this systematic review is the first to investigate gender-by-age patterning of a range of physical morbidity measures across childhood and adolescence in order to assess how strong the evidence is of a generalised or condition/symptom-specific emerging or increasing female excess in physical morbidity over this life stage.

Our descriptive results and meta-regressions provided statistically robust evidence of an emerging or increasing female excess in eight of the 10 physical morbidity measures examined. Prior to conducting our analyses, and in order to help ourselves conceptualise gender-by-age differences in morbidity, we proposed a series of plausible patterns, and our results can be summarised in these terms. A ‘Type 1’ pattern (emerging/increasing female excess) was particularly strong in relation to self-report measures such as self-assessed health and various symptoms, especially headache and abdominal pain. This ‘Type 1’ pattern was also evident in relation to migraine (self-reported but requiring use of recognised diagnostic criteria). In contrast, this pattern was far less robust in relation to epilepsy and there was strongest evidence of an emerging or increasing male excess (‘Type 3’) in relation to type 1 diabetes, both of which were assessed via medical professionals' reports. Thus, gender-by-age differences in measures of physical morbidity varied by both study design and measure.

The proposed ‘Type 1’ patterns were further differentiated in respect of whether the odds of morbidity among females compared to males would start from below, at or above unity, while noting that the specific ages examined will, at least to some extent, determine the pattern observed. While we began with the assumption that, if evident at all, a gender ‘reversal’ was likely to occur in adolescence, the evidence presented here suggests that it occurs at earlier ages in most cases. Among the measures for which there was evidence of a significant increase in the female excess with age, the ‘cross-over age’ was between 6 and 8 years for four health measures (abdominal pain, poor self-assessed health, dizziness and headache) and between 9 and 11 for another four (back pain, migraine, sleeping problems and tiredness).

Research to date suggests that both biological factors, including differing effects of puberty on boys' and girls' health, and social influences, such as the impact of societal gender- and age-related expectations on experiences and reporting of illness, may contribute to gender-by-age patterns in morbidity during the transition from childhood to adolescence (Angold et al., 1999; Haugland et al., 2001; Lien, Dalgard, Heyerdahl, & Bjertness, 2006; Sweeting & West, 2003; Sweeting et al., 2007). There are a number of implications from this review for our understandings of the observed patterns. Firstly, evidence that a female excess was more robust for some measures of physical morbidity than others suggests symptom- or condition-specific explanations and points us towards trying to identify potential mechanisms driving gender-by-age variations in symptom/condition prevalence. Secondly, most ‘reversals’ were estimated to occur prior to the ages at which we would expect puberty to occur, suggesting that this factor might be less important than generally assumed, although some studies (e.g. de Munick Keizer-Schrama & Mul, 2001) report that age at puberty is declining. Thirdly, a female excess was more robust for self-report physical morbidity measures, rather than those based on clinical diagnoses which may have been informed or validated via diagnostic tests. Although self- or other-reporting of symptoms/conditions and medical decisions precede such tests, this finding might be because the ‘gender reversal’ is an artefact of the research methods used, a reflection of greater physical morbidity in females, or a result of under-reporting in males as they get older. Nevertheless, in the absence of self-reported measures of diabetes and epilepsy, and without routine data on consultation rates for a range of symptoms, this hypothesis cannot be further tested.

This review has a number of strengths. First, it was designed and reported based on the PRISMA checklist (Moher et al., 2009). Second, the focus is novel; despite calls for systematic reviewers to widen their focus beyond intervention evaluations, there are relatively few published systematic reviews of epidemiological and other non-intervention studies (Egan, Tannahill, Petticrew, & Thomas, 2008). Third, two search strategies (general and specific) were used across multiple databases to ensure that our search for articles was sufficiently wide yet also adequately sensitive, with the two sets of terms largely identifying different evidence (Egan et al., 2012). Fourth, the data reviewed spanned the transition from early childhood to early-mid adolescence. Fifth, rigorous and systematic methods were used to identify and synthesize the evidence, including checks by at least two reviewers of all extracted data and critical appraisal scores. Sixth, meta-regressions were conducted in order to specifically test the hypothesis of a changing female excess with age in respect of each of the ten morbidity measures. The fact that ‘Type 1 reversals’ were evident, despite the fact that the vast majority of studies included in our review were not specifically designed to examine this issue and so less likely to be subject to publication bias in this respect, is an additional strength. Related to this, although individual datasets may have been underpowered for detection of gender-by-age interactions (since gender ‘reversals’ was not their aim), the strength of a meta-analysis is that study results are combined, thus eliminating the problem of underpowered datasets. In addition, we have found that the hypothesised relative increase in female morbidity tends to be supported by the more robust evidence we identified. Over two thirds (70.2%) of findings from higher scoring studies suggested a Type 1 relationship, of which a third were statistically significant (although higher scoring studies of type 1 diabetes and epilepsy showed non-significant type 3 patterns).

Some limitations should also be highlighted. As with all systematic reviews, there is the potential for publication bias, even if not in respect of gender ‘reversals’, and the studies we accessed were limited to those which were available through the databases we searched. We specifically excluded studies from countries other than the EU, the USA, Canada, Australia and New Zealand, which means our conclusions cannot be extended beyond these countries; findings might differ in, for example, Muslim or Asian countries. Similarly, given that gendered behaviours, including those relating to health, are constrained by what is deemed socially acceptable for boys/girls, men/women within a cultural context at a specific time (Connell, 2012), findings might have differed had we examined older, rather than more contemporary datasets. Our appraisal of the methodological quality of included studies is subject to limitations common to checklist approaches to appraising systematic reviews (Juni, Witschi, Bloch, & Egger, 1999). Furthermore, attempts to appraise study quality are reliant on good reporting of study methods. A further issue is that a large proportion of the evidence for an emerging or increasing female excess came from a small number of large-scale ‘higher-scoring’ studies in which there were statistically significant gender-by-age interactions across (almost) all their self-reported physical morbidity measures (Cavallo et al., 2006; Sweeting & West, 2003; Torsheim et al., 2006).

## Conclusion

For the physical morbidity measures that we reviewed, the balance of evidence broadly pointed towards an emerging or increasing female excess during the transition from childhood to adolescence. Evidence of this pattern was strongest in relation to headache, abdominal pain, tiredness, migraine and self-assessed health, whereas some (although not statistically significant) evidence of an emerging/increasing male excess was found in relation to type 1 diabetes.

Evidence that identifies the age at which gender differentials emerge or increase is important for informing strategies and interventions that aim to prevent or reduce gender inequalities in health. This review provides evidence to suggest that researchers, public health practitioners and policy-makers should recognise mid-late childhood rather than adolescence as a key life stage during which gender inequalities (notably female excess morbidity) emerge. However, our findings also suggest that gender-by-age interactions during this life stage are outcome-specific. To further understandings of the aetiology of gender differences in health, this review highlights a need for good quality longitudinal research which spans childhood and adolescence, i.e. beginning before age 11 which is frequently the youngest age included in such studies, and explores the outcome-specificity of these patterns.

## Figures and Tables

**Table 1:** Results of meta-regressions testing the hypothesis of a changing female excess in each physical morbidity measure with age.

Physical morbidity measure	*B*	SE	*t*	*p*	Odds ratio (95% CI)	Cross-over age (years)
Self-assessed health	0.096	0.022	4.29	<0.001	1.10 (1.05–1.15)	7.9

*Symptoms*						
Abdominal pain	0.100	0.016	6.45	<0.001	1.11 (1.07–1.14)	6.6
Back pain	0.052	0.021	2.41	0.021	1.05 (1.01–1.10)	9.7
Dizziness	0.083	0.028	2.97	0.007	1.09 (1.03–1.15)	8.5
Sleeping problems	0.067	0.014	4.72	<0.001	1.07 (1.04–1.10)	10.3
Tiredness	0.098	0.023	4.36	<0.001	1.10 (1.06–1.15)	11.5
Headache	0.141	0.013	10.76	<0.001	1.15 (1.12–1.18)	8.7

*Conditions*						
Migraine	0.093	0.022	4.27	<0.001	1.10 (1.05–1.15)	9.9
Type 1 diabetes	−0.025	0.019	−1.36	0.183	0.98 (0.94–1.01)	
Epilepsy	0.029	0.014	2.06	0.066	1.03 (1.00–1.06)	

Note: *B* = the coefficient of age on log odds ratio (IRR); SE = standard error.

**Table 2:** Odds (with 95% CI) of poorer self-assessed physical health among females compared with males at each age.

Study	Age	OR (95% CI)
*Categorical self-assessed health studies*		
Cavallo et al. (2006) (XS; HS)^a^	11	1.36 (1.29–1.43)
	13	1.68 (1.60–1.76)
	15	1.97 (1.88–2.06)
Holmberg and Hellberg (2007) (XS)^a^	13–14	1.99 (1.54–2.56)
	15–16	2.51 (1.93–3.25)
	17–18	2.69 (1.99–3.64)
Meland et al. (2007) (XS)^a^	11	1.89 (1.06–3.38)
	13	2.10 (1.39–3.18)
	15	1.78 (1.32–2.40)
Sleskova et al. (2005) (XS)^b^	15–16	1.80 (1.37–2.36)
	17–18	1.82 (1.39–2.38)
Sweeting and West (2003) (L; HS)^c^	11	1.01 (0.86–1.18)
	13	1.30 (1.10–1.53)
	15	1.44 (1.22–1.71)

*Physical health-related quality of life studies*		
Bisegger et al. (2005) (XS) d	9	1.57 (1.13–2.19)
	10	1.46 (1.04–2.06)
	11	1.09 (0.76–1.56)
	12	1.87 (1.33–2.62)
	13	2.13 (1.52–2.99)
	14	2.67 (1.94–3.67)
	15	3.17 (2.24–4.49)
	16	3.12 (2.14–4.54)
	17	3.12 (1.78–5.45)
Jorngarden et al. (2006) (XS)^e^	13–15	0.86 (0.40–1.83)
	16–19	1.22 (0.64–2.34)
Laaksonen et al. (2010) (L)^e^	10	0.94 (0.74–1.18)
	12	1.21 (0.96–1.53)
Palacio-Vieira et al. (2008) (L)^f^		
*Younger cohort*	8–9	0.99 (0.41–2.42)
	11–12	1.04 (0.44–2.51)
*Older cohort*	10–14	1.58 (1.00–2.50)
	13–17	2.55 (1.59–4.10)
Ravens-Sieberer et al. (2008) (XS)^e^	11–13	1.65 (1.27–2.15)
	14–17	3.19 (2.57–3.96)

XS cross-sectional study. L longitudinal study. HS ‘higher-scoring’ study.

a Paper includes percentages ‘poor health’ and precise *N*s required for calculation of ORs and 95% CIs.

b Paper includes percentages ‘poor health’ and sample details (percentages of males and females and in each age group) allowing estimation of *N*s required for calculation of ORs and 95% CIs.

c Precise *N*s required for calculation of ORs and 95% CIs obtained from author.

d Paper includes *N*s and means; SDs obtained from author.

e Paper includes *N*s, means and SDs.

f *N*s, means and SDs obtained from author.

**Table 3:** Odds (with 95% CI) of self-reported abdominal pain, back pain, dizziness, sleeping problems, tiredness and headache among females compared with males at each age.

Study	Age	Abdominal pain	Back pain	Dizziness	Sleeping problems	Tiredness	Headache
		OR (95% CI)	OR (95% CI)				
Cavallo et al. (2006) (XS; HS)^a^	11	1.64 (1.57–1.71)	1.16 (1.10–1.22)	1.18 (1.12–1.24)	1.13 (1.09–1.17)		1.51 (1.45–1.57)
	13	2.00 (1.92–2.09)	1.23 (1.18–1.29)	1.58 (1.50–1.65)	1.36 (1.31–1.41)		1.88 (1.81–1.95)
	15	2.31 (2.20–2.42)	1.28 (1.23–1.34)	1.80 (1.71–1.88)	1.56 (1.50–1.62)		2.70 (2.59–2.81)
Gadin and Hammarstrom (2000) (XS)^a^	9	0.70 (0.34–1.45)			1.16 (0.70–1.95)	1.29 (0.77–2.16)	0.76 (0.39–1.48)
	12	0.89 (0.39–2.04)			1.17 (0.65–2.12)	0.94 (0.57–1.54)	1.69 (0.87–3.26)
Grimmer et al. (2006) (L)^a^	13		1.19 (0.59–2.40)				
	14		1.63 (0.83–3.20)				
	15		2.14 (1.16–3.97)				
	16		1.57 (0.82–2.99)				
	17		2.97 (1.31–6.71)				
Haugland et al. (2001) (XS; HS)^a^							
*Finland*	11	1.63 (1.34–1.99)	0.99 (0.75–1.32)	1.34 (1.04–1.73)	1.10 (0.92–1.31)		1.69 (1.42–2.02)
	13	1.75 (1.38–2.22)	1.30 (0.98–1.71)	1.64 (1.27–2.10)	1.14 (0.93–1.40)		1.96 (1.60–2.39)
	15	1.84 (1.43–2.36)	1.00 (0.78–1.29)	1.42 (1.13–1.79)	1.11 (0.90–1.36)		2.81 (2.28–3.45)
*Norway*	11	1.66 (1.29–2.14)	1.28 (0.90–1.81)	1.45 (1.07–1.98)	1.47 (1.18–1.83)		1.94 (1.49–2.52)
	13	1.48 (1.13–1.93)	1.10 (0.82–1.47)	1.43 (1.07–1.91)	1.17 (0.94–1.46)		1.30 (1.01–1.66)
	15	1.78 (1.34–2.36)	1.06 (0.83–1.37)	1.53 (1.18–2.00)	1.54 (1.23–1.94)		2.19 (1.73–2.78)
*Poland*	11	2.31 (1.74–3.08)	3.29 (1.97–5.48)	2.13 (1.45–3.14)	1.12 (0.87–1.45)		2.53 (1.93–3.30)
	13	2.94 (2.08–4.14)	1.17 (0.79–1.72)	2.56 (1.77–3.72)	1.31 (1.01–1.72)		2.15 (1.65–2.81)
	15	2.33 (1.62–3.37)	1.42 (1.00–2.00)	1.78 (1.33–2.39)	1.80 (1.38–2.33)		3.04 (2.35–3.92)
*Scotland*	11	1.46 (1.17–1.83)	0.87 (0.62–1.22)	1.22 (0.97–1.54)	1.00 (0.83–1.20)		1.31 (1.06–1.61)
	13	1.52 (1.19–1.94)	0.77 (0.57–1.04)	1.26 (0.99–1.60)	1.33 (1.07–1.66)		1.53 (1.23–1.90)
	15	2.40 (1.75–3.29)	1.09 (0.80–1.49)	1.20 (0.92–1.56)	1.50 (1.20–1.89)		2.79 (2.20–3.54)
Heinrich et al. (2009) (XS; HS)^a^	9–10						0.75 (0.56–0.99)
	11–12						0.96 (0.72–1.28)
	13–14						1.12 (0.83–1.50)
Holmberg and Hellberg (2007) (XS)^a^	13	2.86 (1.86–4.39)					2.11 (1.52–2.92)
	14	3.68 (2.30–5.88)					3.06 (2.11–4.44)
	15	4.08 (2.53–6.59)					4.16 (2.86–6.05)
	16	1.96 (1.24–3.10)					4.66 (3.19–6.79)
	17	4.68 (2.90–7.56)					4.59 (3.02–6.99)
Kujala et al. (1999) (XS)^a^	10	1.13 (0.67–1.89)	0.50 (0.19–1.30)				1.38 (0.87–2.18)
	14	2.50 (1.40–4.47)	1.62 (0.82–3.18)				3.38 (2.02–5.68)
	16	3.32 (1.05–10.4)	1.30 (0.44–3.87)				5.00 (1.96–12.7)
Larsson and Sund (2005) (L)^a^	13						2.68 (1.95–3.69)
	14						3.04 (2.21–4.17)
Laurell et al. (2004) (XS)^b^	7						1.10 (0.42–2.86)
	8						1.16 (0.55–2.44)
	9						0.81 (0.42–1.58)
	10						0.97 (0.49–1.92)
	11						1.45 (0.78–2.70)
	12						1.95 (1.08–3.51)
	13						1.46 (0.75–2.87)
	14						3.33 (1.75–6.36)
	15						3.77 (2.02–7.03)
Leonardsson-Hellgren et al. (2001) (XS)^a^	13–14						0.87 (0.43–1.75)
	14–15						0.84 (0.32–2.17)
	15–16						1.37 (0.50–3.78)
Lundqvist et al. (2006) (XS)^b^	7						0.62 (0.34–1.12)
	8						1.34 (0.88–2.02)
	9						1.01 (0.67–1.53)
	10						0.83 (0.55–1.24)
	11						1.79 (1.19–2.68)
	12						1.22 (0.81–1.83)
Mortimer et al. (1993, 1992) (XS)^a^	5–7	1.04 (0.54–2.01)					1.05 (0.63–1.74)
	9–11	1.05 (0.40–2.71)					1.03 (0.64–1.67)
Ostberg et al. (2006) (XS; HS)^b^	10	1.84 (1.28–2.65)			1.00 (0.74–1.35)		1.90 (1.32–2.73)
	11	1.70 (1.17–2.49)			0.82 (0.60–1.11)		1.77 (1.25–2.50)
	12	2.65 (1.78–3.93)			1.35 (0.99–1.86)		1.54 (1.09–2.18)
	13	1.58 (1.07–2.31)			1.03 (0.75–1.41)		1.47 (1.05–2.06)
	14	2.14 (1.35–3.39)			1.13 (0.81–1.59)		2.40 (1.67–3.46)
	15	1.47 (0.95–2.28)			1.43 (1.00–2.06)		2.19 (1.52–3.14)
	16	3.05 (1.91–4.86)			1.21 (0.84–1.73)		3.00 (2.05–4.38)
	17	3.10 (1.83–5.24)			1.10 (0.76–1.59)		2.30 (1.55–3.41)
Petersen et al. (2003) (XS; HS)^a^	6	0.53 (0.21–1.35)	2.06 (0.18–23.2)			0.72 (0.34–1.50)	0.59 (0.14–2.57)
	7	1.31 (0.60–2.84)	0.38 (0.04–3.71)			0.83 (0.39–1.79)	1.19 (0.46–3.04)
	8	1.36 (0.62–2.95)	0.70 (0.11–4.28)			0.67 (0.31–1.47)	1.15 (0.56–2.34)
	9	2.14 (0.91–5.04)	0.87 (0.24–3.13)			0.82 (0.41–1.61)	2.30 (1.01–5.20)
	10	1.55 (0.70–3.44)	0.76 (0.23–2.52)			0.99 (0.49–2.00)	1.13 (0.57–2.24)
	11	2.03 (0.94–4.38)	0.91 (0.38–2.16)			0.97 (0.53–1.77)	1.67 (0.87–3.22)
	12	1.30 (0.61–2.78)	1.95 (0.82–4.66)			1.00 (0.51–1.93)	1.38 (0.70–2.73)
Santinello et al. (2009) (XS; HS)a,e	11						1.23 (1.00–1.53)
	13						2.15 (1.75–2.64)
	15						2.91 (2.29–3.68)
Sleskova et al. (2005) (XS)^d^	15–16		2.20 (1.56–3.11)	2.28 (1.39–3.73)		1.76 (1.35–2.28)	2.03 (1.50–2.74)
	17–18		2.29 (1.69–3.10)	4.31 (2.47–7.53)		2.09 (1.61–2.70)	4.18 (2.98–5.86)
Sundblad et al. (2007) (XS; HS)^a^	9	1.39 (0.77–2.51)			0.68 (0.42–1.11)	0.37 (0.19–0.73)	1.47 (0.80–2.69)
	12	1.90 (0.99–3.63)			0.98 (0.59–1.64)	1.23 (0.75–2.01)	2.28 (1.42–3.66)
	15	2.70 (1.42–5.10)			1.83 (1.12–2.99)	1.25 (0.89–1.76)	2.87 (1.82–4.52)
Sweeting and West (2003) (L; HS)^b^	11	1.48 (1.26–1.74)		0.82 (0.67–1.00)	0.89 (0.76–1.04)		1.49 (1.28–1.75)
	13	2.07 (1.74–2.47)		1.25 (1.04–1.50)	1.14 (0.96–1.35)		1.48 (1.25–1.76)
	15	3.15 (2.62–3.80)		1.68 (1.40–2.02)	1.69 (1.42–2.01)		2.41 (1.98–2.92)
Torsheim et al. (2006) (XS; HS)^c^	11	1.86 (1.69–2.05)	1.25 (1.14–1.37)	1.41 (1.26–1.58)	1.11 (1.04–1.18)		1.58 (1.46–1.71)
	13	2.29 (2.02–2.59)	1.29 (1.19–1.40)	1.83 (1.62–2.06)	1.36 (1.26–1.47)		2.01 (1.84–2.19)
	15	2.67 (2.33–3.06)	1.34 (1.22–1.47)	1.79 (1.59–2.01)	1.57 (1.46–1.69)		2.90 (2.64–3.18)
Wedderkopp et al. (2001) (XS)^a^	8–10		0.34 (0.13–0.90)				
	14–16		1.22 (0.70–2.11)				
Rhee et al. (2005) (XS)^f^	11–21	Female excess at all ages		Female excess at all ages		Female excess increased significantly with age	Female excess increased significantly with age

XS cross-sectional study. L longitudinal study. HS ‘higher-scoring’ study.

a Paper includes percentages with symptom and precise *N*s required for calculation of ORs and 95% CIs.

b Precise *N*s required for calculation of ORs and 95% CIs obtained from author.

c Paper includes ORs and 95% CIs of symptom among females compared with males.

d Paper includes percentages ‘poor health’ and sample details (percentages of males and females and in each age group) allowing estimation of *N*s required for calculation of ORs and 95% CIs.

e Excluded from meta-analysis (data included in Cavallo et al., 2006).

f Descriptive results from text of paper; further data unavailable; excluded from meta-analyses.

**Table 4:** Odds (with 95% CI) of migraine among females compared with males at each age.

Study	Ages	OR (95% CI)
Bigal et al. (2007) (XS)^b^	12	0.93 (0.66–1.32)
	13	1.23 (0.89–1.70)
	14	1.16 (0.86–1.57)
	15	1.57 (1.17–2.10)
	16	2.22 (1.62–3.05)
	17	2.56 (1.95–3.35)
Gordon et al. (2004) (XS)^c^	12–14	1.67
	15–19	2.45
Heinrich et al. (2009) (XS; HS)^a^	9–10	1.12 (0.77–1.61)
	11–12	0.93 (0.68–1.29)
	13–14	1.13 (0.84–1.53)
Laurell et al. (2004) (XS)^d^	7–9	0.70 (0.30–1.64)
	10–12	1.50 (0.86–2.62)
	13–15	1.48 (0.89–2.45)
Mavromichalis et al. (1999) (XS; HS)^a^	4–6	0.54 (0.13–2.18)
	7–9	1.03 (0.63–1.66)
	10–12	1.68 (1.10–2.56)
	13–15	2.00 (1.00–3.98)

XS cross-sectional study. HS ‘higher-scoring’ study.

a Paper includes percentages with migraine and precise *N*s required for calculation of ORs and 95% CIs.

b Paper includes percentages with migraine and sample details (percentages of males and females an in each age group) allowing estimation of *N*s required for calculation of ORs and 95% CIs.

c Paper includes percentages; *N*s required for calculation of 95% CIs unavailable.

d Paper includes percentages; *N*s obtained from author.

**Table 5:** Incidence rate ratios (with 95% CIs) of diabetes and epilepsy among females compared with males at each age.

Study	Ages	Diabetes	Epilepsy
		IRR (95% CI)	IRR (95% CI)
Carle et al. (2004) (RD; HS)^a^			
*Northern Italy*	5–9	0.84 (0.63–1.12)	
	10–14	0.76 (0.58–1.00)	
*Central Italy*	5–9	0.95 (0.78–1.16)	
	10–14	0.86 (0.71–1.05)	
*Southern Italy*	5–9	1.21 (0.95–1.54)	
	10–14	0.76 (0.59–0.98)	
*Sardegna*	5–9	0.79 (0.64–0.98)	
	10–14	0.61 (0.50–0.75)	
Casu et al. (2004) (RD)^b^	4	0.83 (0.49–1.42)	
	5	0.94 (0.58–1.53)	
	6	1.02 (0.67–1.57)	
	7	0.49 (0.32–0.77)	
	8	0.56 (0.35–0.90)	
	9	0.87 (0.60–1.25)	
	10	0.66 (0.45–0.96)	
	11	0.84 (0.57–1.24)	
	12	0.36 (0.24–0.55)	
	13	0.55 (0.37–0.81)	
	14	0.49 (0.31–0.77)	
Cinek et al. (2000) (RD)^b^	4	0.94 (0.62–1.43)	
	5	1.23 (0.83–1.82)	
	6	0.96 (0.65–1.40)	
	7	0.89 (0.61–1.29)	
	8	1.21 (0.83–1.74)	
	9	1.40 (1.00–1.97)	
	10	1.42 (1.02–1.96)	
	11	1.29 (0.93–1.78)	
	12	1.33 (0.98–1.80)	
	13	0.72 (0.53–0.98)	
	14	0.89 (0.65–1.23)	
Cotellessa et al. (2003) (RD)^a^	5–9	0.60 (0.36–1.01)	
	10–14	0.73 (0.47–1.13)	
Karvonen et al. (1999) (RD)^c^	5–9	0.98	
	10–14	0.82	
Michalkova et al. (1995) (RD)^d^	5–9	1.15 (0.63–2.07)	
	10–14	1.69 (0.91–3.16)	
Skordis et al. (2002) (RD)^b^	5–6	0.60 (0.26–1.37)	
	7–8	1.17 (0.54–2.52)	
	9–10	1.60 (0.84–3.05)	
	11–12	1.33 (0.68–2.60)	
	13–14	0.73 (0.34–1.60)	
Beilmann et al. (1999) (RD, HS)^a^	5–9		1.40 (0.78–2.51)
	10–14		0.70 (0.38–1.28)
	15–19		0.80 (0.22–2.92)
Christensen et al. (2007) (RD)^b^	4–5		0.85 (0.75–0.97)
	6–7		0.81 (0.72–0.92)
	8–9		0.78 (0.69–0.89)
	10–11		0.97 (0.85–1.12)
	12–13		1.09 (0.93–1.27)
	14–15		1.27 (1.09–1.47)
	16–17		1.15 (0.99–1.34)
Freitag et al. (2001) (RD)^e^	5–9		2.33 (0.60–9.03)
	10–14		0.83 (0.25–2.73)

RD study based on routinely collected data. HS ‘higher-scoring’ study.

a Paper includes IRRs and 95% CIs of condition among females compared with males.

b Precise *N*s required for calculation of IRRs and 95% CIs obtained from author.

c Paper includes incidence rates; *N*s required for calculation of 95% CIs unavailable.

d Paper includes incidence *N*s and rates, allowing calculation of *N*s required for calculation of 95% CIs.

e Paper includes graphed incidence rates and total population at risk, which when split in males and females allowed calculation of *N*s required for calculation of 95% CIs.
