# Supplementary material for: How robust is the evidence of an emerging or increasing female excess in physical morbidity between childhood and adolescence? Results of a systematic literature review and meta-analyses
Source: Soc Sci Med. 2013 Feb;78:96–112. doi: 10.1016/j.socscimed.2012.11.039 (PMC3566587; doi:10.1016/j.socscimed.2012.11.039)
Supplement: Supplementary file 1 [file mmc1.docx]

**Supplementary Table 1: Summary of studies included in review**

| **First author and date of publication** | **Study name**  **Setting**  **Source** | **Methods** | **Date of data collection** | **Sample details** | **Extracted health outcome** | **Gender-by-age differences in morbidity?** | **Study score*** |
| --- | --- | --- | --- | --- | --- | --- | --- |
| **Longitudinal studies** | | | | | | | |
| Grimmer et al. (2006) | Not stated  Australia (Adelaide)  School | Self-complete questionnaire | 1999, 2000, 2001, 2002 and 2003 | Initial sample = 526  Achieved sample = 434 (baseline); 315 (wave 2); 300 (wave 3); 244 (wave 4); 174 (wave 5)  Response rate = 82.5% (baseline); 72.2% (of baseline at wave 2); 69.1% (of baseline at wave 3); 56.2% (of baseline at wave 4); 40.1% (of baseline at wave 5)  Ages compared: 13, 14, 15, 16, 17 | Back ache  (low back pain in past week) | Type 1 | 6 |
| Laaksonen et al. (2010) | Turku Schools on the Move project  Finland  School | Self-complete questionnaire | 2004 and 2006 | Initial sample = 1,346  Achieved sample = 1,094 (baseline) ; 986 (follow-up)  Response rate = 81% (baseline); 90.1% (of baseline at follow-up)  Ages compared: 10, 12 | General physical health  (physical subscale from Finnish version of Pediatric Quality of life Inventory) | Type 2 | 10 |
| Larsson & Sund (2005) | Not stated  Norway (two central counties)  School | Self-complete questionnaire | 1998 and 1999 | Initial sample = 2,792  Achieved sample = 2,465 (baseline); 2,355 (follow-up)  Response rate = 88.3% (baseline); 95.5% (of baseline at follow-up)  Ages compared: 13.7, 14.9 | Headache  (headaches more than once a week) | Type 1 | 10 |
| Palacio-Vieira et al. (2008) | European KIDSCREEN study  Spain  General  population | Self-complete (postal) questionnaire | 2003 and 2006 | Initial sample = 1,956 (households)  Achieved sample = 926 (baseline); 454 (follow-up)  Response rate = 47.2% (baseline); 54.0% (of baseline at follow-up)  Ages compared: 11-12, 13-17, 18-21 | General physical health  (physical well-being dimension from KIDSCREEN-52 health-related quality of life measure) | Type 1 | 7 |
| Sweeting & West (2003) | West of Scotland 11 to 16: Teenage Health  Scotland (Glasgow)  School | Self-complete Questionnaire | 1994/5, 1996 and 1999 | Initial sample = 2,793  Achieved sample = 2,586 (wave 1); 2,371 (wave 2); 2,196 (wave 3)  Response rate = 93% (wave 1); 92% (of baseline at wave 2); 85% (of baseline at wave 3)  Ages compared: 11, 13, 15 | General physical health  (past 12 months self assessed health ‘fairly’/’not good’ vs ‘good’)  Abdominal pain  (past month sickness or stomach ache)  Dizziness  (past month dizzy or faint)  Sleeping problems  (past month difficulty getting to sleep)  Headache  (past month headache) | Type 1**†**  Type 1**†**  Type 1**†**  Type 1**†**  Type 1**†** | 12 |
| **Repeat cross-sectional/Cross-sectional studies** | | | | | | | |
| Bigal et al. (2007) | American Migraine Prevalence and Prevention Project  USA  General population | Self-complete questionnaire | Not stated | Initial sample = 32,015 adolescents (in 120,000 households)  Achieved sample = 18,714  Response rate = 58.4%  Ages compared: 12, 13, 14, 15, 16, 17, 18, 19 | Migraine (past year prevalence) | Type 1 | 6 |
| Bisegger et al. (2005) | Kidscreen 52 Pilot Study  Austria, France, Germany, Spain, Switzerland, UK (schools);  Netherlands (general population) | Self-complete questionnaire | Not stated | Initial sample = 7,014  Achieved sample = 3,710  Response rate = 56.7%  Ages compared: 9, 10, 11, 12, 13, 14, 15, 16, 17 | General physical health  (physical well-being dimension from KIDSCREEN-52 health-related quality of life measure) | Type 1 | 7 |
| Cavallo et al. (2006) | HBSC International Collaboration Study  Europe and North America  School | Self-complete questionnaire | 2001/2002 | Initial sample = not stated  Achieved sample = approx. 160,000  Response rate = not stated  Ages compared:11, 13, 15 | General physical health  (current self-assessed health ‘poor’/’fair’ vs ‘good’/’excellent’)  Abdominal pain (stomach ache at least weekly in past 6 months)  Backache  (at least weekly in past 6 months)  Dizzinesss  (at least weekly in past 6 months)  Sleeping problems  (difficulties in sleeping at least weekly in past 6 months)  Headache  (at least weekly in past 6 months) | Type 1**†**  Type 1**†**  Type 1**†**  Type 1**†**  Type 1**†**  Type 1**†** | 9 |
| Gadin & Hammarstrom (2000) | Not stated  Sweden (north)  School | Self-complete questionnaire | 1994 | Initial sample = not stated  Achieved sample = 538  Response rate = assume 100% (‘all students in the grades under study were included … no lack of information due to non-response’)  Ages compared: 9, 12 | Abdominal pain (frequency = always/often)  Difficulty falling asleep  (always/often)  Tired  (always/often)  Headache  (always/often) | Type 1  Type 2  Type 3  Type 1 | 8 |
| Gordon et al. (2004) | Canadian National Population Health Survey 1996-97  Canada  General population | Structured interview | 1996-1997 | Initial sample = 173,216 (‘eligible participants’, aged 12+ years)  Achieved sample = 173,012  Response rate = 99%  Ages compared: 12-14, 15-19, ≥20 (subset of data) | Migraine  (current prevalence ) | Type 1 | 7 |
| Haugland et al. (2001) | HBSC (Finland, Norway, Poland and Scotland data)  Finland, Norway, Poland and Scotland  School | Self-complete questionnaire | 1993-1994 | Initial sample = not stated  Achieved sample = 20,324 (all countries)  Response rate =  Finland (93%); Norway (82%); Poland (91%); Scotland (78%)  Ages compared: 11, 13, 15 | Abdominal pain  (stomach ache at least weekly in past 6 months)  Backache  (at least weekly in past 6 months)  Dizziness  (at least weekly in past 6 months)  Sleeping problems (difficulties in sleeping at least weekly in past 6 months)  Headache  (at least weekly in past 6 months) | Finland: Type 1  Norway: Type 4  Poland : Type 4  Scotland: Type 1  Finland: Type 4  Norway: Type 3  Poland: Type 3  Scotland: Type 4  Finland: Type 4  Norway: Type 2  Poland: Type 4  Scotland: Type 2  Finland: Type 2  Norway: Type 4  Poland: Type 1  Scotland: Type 1  Finland: Type 1**†**  Norway: Type 4  Poland: Type 4  Scotland: Type 1**†** | 11 |
| Heinrich et al. (2009) | Children, Adolescents and Headache  Germany (southern Lower Saxony and Hannover)  General population | Self-complete (postal) questionnaire | 2003/2004 | Initial sample = 6,400 households with 9-14 year old children  Achieved sample = 4,043  Response rate = 63.2%  Ages compared: 9-10, 11-12, 13-14 | Headache  (6-month prevalence of tension-type headache)  Migraine  (6-month prevalence of ICHD-II [2004] headache) | Type 1  Type 2 | 9 |
| Holmberg & Hellberg (2007) | Not stated  Sweden (one central town)  School | Self-complete questionnaire (Q90) | 2004 | Initial sample = 3,812  Achieved sample = 3,186  Response rate = 83.6%  Ages compared: 13, 14, 15, 16, 17, 18 | General physical health  (do not ‘feel healthy’)  Abdominal pain  (past week prevalence)  Headache  (past week prevalence) | Type 1  Type 4  Type 1**†** | 8 |
| Jorngarden et al. (2006) | Not stated  Sweden  General population | Self-complete (postal) questionnaire and telephone interview (SF36) | 2005 | Initial sample = 840  Achieved sample = 585  Response rate = 69.6%  Ages compared: 13-15, 16-19, 20-23 | General physical health  (physical functioning dimension from SF-36 health-related quality of life measure) | Type 2 | 5 |
| Kujala et al. (1999) | Not stated  Finland  School | Self-complete questionnaire | 1993 | Initial sample = not stated  Achieved sample = 698  Response rate = not stated  Ages compared: 10, 14, 16 | Abdominal pain  (past year prevalence)  Backache  (past year prevalence of lower back pain)  Headache  (past year prevalence) | Type 1  Type 4  Type 1 | 7 |
| Laurell et al. (2004) | Not stated  Sweden (Uppsala)  School | Self-complete questionnaire (completed at home) | 1997 | Initial sample = 1,850  Achieved sample = 1,371  Response rate = 74.1%  Ages compared: 7-9, 10-12, 13-15 | Headache  (past year prevalence of tension-type headache)  Migraine  (past-year prevalence of migraine with strict or modified IHS criteria) | Type 1  Type 1 | 8 |
| Leonardsson-Hellgren et al. (2001) | Not stated  Sweden (Skovde)  School | Self-complete questionnaire | Not stated | Initial sample = 344  Achieved sample = 344  Response rate = 100%  Ages compared: 13-14, 14-15, 15-16 | Headache  (at least weekly) | Type 1 | 5 |
| Lundqvist et al. (2006) | Not stated  Norway (Oslo)  School | Self-complete diaries completed each school day for a 6-week period (total 30 days) | Not stated | Initial sample = not stated  Achieved sample = 2,174  Response rate = 94% of those issued with diaries (a subset of pupils in questionnaire study with an 85% response rate)  Ages compared: 7, 8, 9, 10, 11, 12 | Headache  (number of days with headache) | Type 2 | 8 |
| Mavromichalis et al. (1999) | Not stated  Greece (Thesaloniki)  School | Self-complete questionnaire (completed at home) | 1997 | Initial sample = 4,000  Achieved sample = 3,509  Response rate = 87.7%  Ages compared: 4-6, 7-9, 10-12, 13-15 | Migraine  (past year prevalence of IHS diagnostic criteria for migraine) | Type 1 | 9 |
| Meland et al. (2007) | HBSC  Norway  School | Self-complete questionnaire | Dec 1997 | Initial sample = 6,613  Achieved sample = 5,026  Response rate = 76%  Ages compared: 11, 13, 15 | General physical health  (self-assessed ‘not very healthy’ vs ‘very’/’quite healthy’) | Type 4 | 8 |
| Mortimer et al. (1992) | Not stated  UK  GP practice | Structured interview | Not stated | Initial sample = 1,104  Achieved sample = 1,083  Response rate = 98.1%  Ages compared: 4, 5, 6, 7, 8, 9, 10, 11 | Headache  (last year prevalence) | Type 1 | 6 |
| Mortimer et al. (1993) | Not stated (same data as above)  UK  GP practice | Structured  interview | Not stated | Initial sample = 1,104  Achieved sample = 1,083  Response rate = 98.1%  Ages compared: 3-5, 5-7, 7-9, 9-11 | Abdominal pain  (past year recurrent abdominal pain) | Type 2 | 5 |
| Ostberg et al. (2006) | Level of Living Survey (Sweden) and Survey of Living Conditions (ULF)  Sweden  General population | Self-complete (audio) questionnaire | 2000 (Level of Living Survey) and 2001-2003 (Survey of Living Conditions) | Initial sample = 6,573  Achieved sample = 5,390  Response rate = 82%  Ages compared: 10, 11, 12, 13, 14, 15, 16, 17, 18 | Abdominal pain  (stomach ache weekly or more in past 6 months))  Sleeping problems  (difficulty falling asleep weekly or more in past 6 months)  Headache  (weekly or more in past 6 months) | Type 1  Type 4  Type 1 | 9 |
| Petersen et al. (2003) | Not stated  Sweden (Umea)  School | Self-complete questionnaire (question from HBSC) | 2001 | Initial sample = 1,155  Achieved sample = 1,121  Response rate = 97%  Ages compared: 6, 7, 8, 9, 10, 11, 12 | Abdominal pain  (stomach ache at least weekly in past 6 months)  Backache  (at least weekly in past 6 months)  Tiredness  (tired when starting school at least weekly in past 6 months)  Headache  (at least weekly in past 6 months) | Type 4  Type 4  Type 4  Type 4 | 9 |
| Ravens-Sieberer et al. (2008) | BELLA Study (sub-sample of German National Health Interview and Examination Survey for Children and Adolescents (KiGGS))  Germany  General population | Self-complete questionnaire (KINDL-R) | 2003- 2006 | Initial sample = 4,199 (families with 7-17 year old children)  Achieved sample = 1,895 (participants)  Response rate = 68%  Ages compared: 7-10, 11-13, 14-17 (subset of data) | General physical health  (physical subscale from KINDL-R health related quality of life measure) | Type 1 | 8 |
| Rhee et al. (2005) | National Longitudinal Study of Adolescent Health (Add Health)  U.S  School | Structured interview | 1994-1995 | Initial sample = not stated  Achieved sample = 20,745  Response rate = not stated  Ages compared: 11, 12, 13, 14, 15, 16, 17, 18, 19, 20, 21 | Abdominal pain  (stomach ache weekly or more in past 12-months)  Dizziness  (dizziness weekly or more in past 12 months)  Tiredness  (fatigue weekly or more in past 12 months)  Headache  (weekly or more in past 12 months) | Type 2  Type 2  Type 1  Type 1 | 8 |
| Santinello et al. (2009) | HBSC  Italy  School | Self-complete questionnaire | 2002 | Initial sample = not stated  Achieved sample = 4,386  Response rate = 77.4% of selected schools  Ages compared: 11, 13, 15 | Headache  (weekly or more in past 6 months) | Type 1**†** | 9 |
| Sleskova et al. (2005) | Not stated  Slovakia  School | Self-complete questionnaire | 2002 | Initial sample = 3,842  Achieved sample = 2,836  Response rate = 97.5% for younger two cohorts; 45.5% for oldest group.  Ages compared: 15.9, 17.8 | General physical health  (self-assessed health ‘good’/’fairly good’/’bad’ vs ‘excellent’/’very good’)  Backache  (three times or more in past month)  Dizziness  (three times or more in past month)  Tiredness  (get up feeling tired three times or more in past month)  Headache  (three times or more in past month) | Type 2  Type 2  Type 1  Type 2  Type 1 | 7 |
| Sundblad et al. (2007) | Not stated  Sweden  School | Self-complete questionnaire | 2002 | Initial sample = 1,975  Achieved sample = 1,908  Response rate = 96.6%  Ages compared: 9, 12, 15 | Abdominal pain  (weekly or more in past 10-14 weeks)  Sleeping problems (weekly or more in past 10-14 weeks)  Tiredness  (weekly or more in past 10-14 weeks)  Headache  (weekly or more in past 10-14 weeks) | Type 1  Type 1  Type 1**†**  Type 1 | 11 |
| Torsheim et al. (2006) | HBSC  Europe and North America (29 countries)  School | Self-complete questionnaire (HBSC) | 1997-1998 | Initial sample = not stated  Achieved sample = 125,732  Response rate = ‘The response rate (RR) at the level of school was in general high, with a majority of countries above 80%’.  Ages compared: 11, 13, 15 | Abdominal pain  (stomach ache at least weekly in past 6 months)  Backache  (at least weekly in past 6 months)  Dizziness  (at least weekly in past 6 months)  Sleeping problems  (difficulties in sleeping at least weekly in past 6 months)  Headache  (at least weekly in past 6 months) | Type 1**†**  Type 1  Type 1**†**  Type 1**†**  Type 1**†** | 9 |
| Wedderkopp et al. (2001) | Not stated  Denmark  School | Structured interview | 1997-1998 | Initial sample = 806 (sub-sampled from a larger study which had a 75% response rate)  Achieved sample = 806  Response rate = 100%  Ages compared: 8-10, 14-16 | Backache  (past month prevalence of low back pain) | Type 1 | 8 |
| **Routine data studies** | | | | | | | |
| Beilmann et al. (1999) | Not stated  Estonia  Clinic/hospital | Physician examination records | 1995-1997 | N = 161,202 (mean population (aged 1 month and 19 years) between 1st Jan 1995 and 31^st^ Dec 1997 )  Cases = 216 new cases (110M/ 106F)  Completeness =  not stated but ‘the possibility of treating patients with epilepsy outside the study area is minimal’  Ages compared: 0-4, 5-9, 10-14, 15-19 | Epilepsy  (incidence) | Type 3 | 12 |
| Carle et al. (2004) | RIDI Study Group  Italy  Clinic/hospital and other (e.g. prescription registries) | Registries of Type 1 diabetes | 1990-1999 | N = 15,718,296 (at risk population (all ages) covered by registries)  Cases = 3,602 new cases aged 0-14 years (2016M/1586F)  Completeness = 90.7 – 99%  Ages compared: 0-4, 5-9, 10-14 | Diabetes  (incidence) | North: Type 3  Central: Type 3  South: Type 3  Sardegna: Type 3 | 12 |
| Casu et al. (2004) | Not stated  Sardinia  Clinic/hospital | Sardinian Type 1 diabetes register | 1989-1999 | N = 317,571 (Sardinian population aged <15 years in 1989); 240,251 (Sardinian population aged <15 years in 1999). Population figures are not given for 1990-1998.  Cases = 1,214 new cases (M/F not stated)  Completeness = 91%  Ages compared: 0-4, 5-9, 10-14 | Diabetes  (incidence) | Type 3 | 10 |
| Christensen et al. (2007) | Not stated  Denmark  General population and clinic/hospital | Danish Civil Registration System and Danish National Hospital Register | 1977-2002 | N = 6,543,341 (all persons born in Denmark and who were alive and resident in Denmark at least 1 day between 1 Jan 1977 and 31 Dec 2002 (all ages))  Cases = 88,616 new cases (M/F not stated)  Completeness = not stated  Ages compared: 0, 10, 20, 30, 40, 50, 60, 70, 80, 90 | Epilepsy  (incidence) | Type 1 | 9 |
| Cinek et al. (2000) | Not stated  Czech Republic  Clinic/hospital and general population | Czech Childhood Diabetes Registry network and  Association of Parents and Friends of Diabetic Children database | 1990-1997 | N = 15, 849, 731 (population aged 0-14 years between 1990-97)  Cases = 1,604 new cases (814M/790F)  Completeness = 99.7%  Ages compares: 0-4, 5-9, 10-14 | Diabetes  (incidence) | Type 2 | 11 |
| Cotellessa et al. (2003) | Not stated  Italy  Clinic/hospital | Physician examination records | 1989-1998 | N = 1,754,418 (population aged 0-14 years between 1989-98)  Cases = 219 new cases (126M/93F)  Completeness = 98.16%  Ages compared: 0-4, 5-9, 10-14 | Diabetes  (incidence) | Type 1 | 11 |
| Freitag et al. (2001) | Not stated  Germany (Mannheim and Heidelberg)  Clinic/hospital | Physician examination records | 1999–2000 | N = 59,647 (population aged 1 month- 14 years in Mannheim and Heidelberg between 1^st^ July 1999 and 30^th^ June 2000)  Cases = 36 new cases (M/F not stated)  Completeness = not stated  Ages compared: 1-4, 5-10, 11-14 | Epilepsy  (incidence) | Type 3 | 10 |
| Karvonen et al. (1999) | Not stated  Finland  General population and clinic/hospital | Central Drug Registry of the Social Insurance Institution and the Prospective Childhood Diabetes Registry at National Public Health Institute, Helsinki | 1965-1996 | N = 1,239,103 (population aged 0-14 in 1965); 971,770 (population aged 0-14 in 1996). Population figures are not given for 1966-1995.  Cases = 9,808 new cases (M/F not stated)  Completeness = ‘virtually 100%’  Ages compared: 1-4, 5-9, 10-14 | Diabetes  (incidence) | Type 3 | 11 |
| Michalkova et al. (1995) | Not stated  Slovakia  Clinic/hospital | Physician examination records | 1985-1992 | N = 1,363,499 (mean population aged 0-14 between 1985-1992)  Cases = new cases 754 (369M/385F)  Completeness = 95%  Ages compared: 0-4, 5-9, 10-14 | Diabetes  (incidence) | Type 1 | 11 |
| Skordis et al. (2002) | Not stated  Cyprus  Clinic/hospital | Physician examination records | 1990-2000 | N = 155,777 (mean population aged 0-15 years between 1990-2000)  Cases = 194 (99M/95F)  Completeness = not stated  Ages compared: 0-4, 5-9, 10-14 | Diabetes  (incidence) | Type 4 | 11 |

* An additional criterion was used to score longitudinal studies (attrition rate). As a result the maximum score for longitudinal studies was 14, as opposed to 12 for repeat cross-sectional/cross-sectional and routine data studies.

† Non-overlapping 95% confidence intervals.
